# Proceedings of the 2nd BEAT-PCD conference and 3rd PCD training school: part 1

**DOI:** 10.1186/s12919-018-0098-9

**Published:** 2018-03-05

**Authors:** Florian Halbeisen, Claire Hogg, Mikkel C. Alanin, Zuzanna Bukowy-Bieryllo, Francisco Dasi, Julie Duncan, Amanda Friend, Myrofora Goutaki, Claire Jackson, Victoria Keenan, Amanda Harris, Robert A. Hirst, Philipp Latzin, Gemma Marsh, Kim Nielsen, Dominic Norris, Daniel Pellicer, Ana Reula, Bruna Rubbo, Nisreen Rumman, Amelia Shoemark, Woolf T. Walker, Claudia E. Kuehni, Jane S. Lucas

**Affiliations:** 1 Institute of Social and Preventive Medicine, University of Bern, Bern, Switzerland; 2 Primary Ciliary Dyskinesia Centre, Departments of Paediatrics and Paediatric Respiratory Medicine, Imperial College and Royal Brompton Hospital, London, UK; 3 Department of Otolaryngology - Head and Neck Surgery and Audiology, Copenhagen University Hospital, Copenhagen, Denmark; 4 Department of Molecular and Clinical Genetics, Institute of Human Genetics Polish Academy of Sciences, Poznań, Poland; 5 Department of Physiology, Faculty of Medicine, Universitat de Valencia, Valencia, Spain; 6 UCIM Department, Instituto de Investigación Sanitaria INCLIVA, Valencia, Spain; 7 Primary Ciliary Dyskinesia Centre, Departments of Paediatrics and Paediatric Respiratory Medicine, Imperial College and Royal Brompton Hospital, London, UK; 8 Primary Ciliary Dyskinesia Centre, NIHR Southampton Biomedical Research Centre, University of Southampton and University Hospital Southampton NHS Foundation Trust, Southampton, UK; 9 Centre for PCD Diagnosis and Research, Department of Infection, Immunity and Inflammation, University of Leicester, Robert Kilpatrick Clinical Sciences Building, Leicester Royal Infirmary, Leicester, UK; 10 Paediatric Respiratory Medicine, University Children’s Hospital of Bern, University of Bern, Bern, Switzerland; 11 Danish PCD & chILD Centre, CF Centre Copenhagen Paediatric Pulmonary Service, ERN Accredited for PCD and CF Health Care, Department of Paediatrics and Adolescent Medicine, Copenhagen University Hospital, Rigshospitalet, Copenhagen, Denmark; 12 Mammalian Genetics Unit, MRC Harwell Institute, Harwell Campus, Oxfordshire, UK; 13 Pediatric Department, Makassed Hospital, East Jerusalem, Palestine; 14 School of Medicine, University of Dundee, Dundee, UK

## Abstract

Primary ciliary dyskinesia (PCD) is a rare heterogenous condition that causes progressive suppurative lung disease, chronic rhinosinusitis, chronic otitis media, infertility and abnormal situs. ‘Better Experimental Approaches to Treat Primary Ciliary Dyskinesia’ (BEAT-PCD) is a network of scientists and clinicians coordinating research from basic science through to clinical care with the intention of developing treatments and diagnostics that lead to improved long-term outcomes for patients. BEAT-PCD activities are supported by EU funded COST Action (BM1407). The second BEAT-PCD conference, and third PCD training school were held jointly in April 2017 in Valencia, Spain. Presentations and workshops focussed on advancing the knowledge and skills relating to PCD in: basic science, epidemiology, diagnostic testing, clinical management and clinical trials. The multidisciplinary conference provided an interactive platform for exchanging ideas through a program of lectures, poster presentations, breakout sessions and workshops. Three working groups met to plan consensus statements. Progress with BEAT-PCD projects was shared and new collaborations were fostered. In this report, we summarize the meeting, highlighting developments made during the meeting.

**Electronic supplementary material:**

The online version of this article (10.1186/s12919-018-0098-9) contains supplementary material, which is available to authorized users.

## Introduction

Primary ciliary dyskinesia (PCD) is a rare syndrome characterized by impaired mucociliary clearance due to abnormal ciliary function. It is usually inherited as an autosomal recessive condition, but X-linked inheritance has been described; mutations in > 35 genes have been reported to cause PCD, and more genes remain to be found [1–3]. Clinical manifestations are caused by impaired mucociliary clearance and include recurrent lower and upper respiratory tract symptoms which present soon after birth [4, 5]. Pulmonary disease is progressive, with recurrent infections leading to bronchiectasis and impaired lung function. Sperm flagella have a similar ultrastructure to cilia and male infertility is well described but the prevalence is unclear due to lack of data. Motile embryonic nodal cilia establish left-right asymmetry and nearly half of PCD patients exhibit situs inversus and 6–12% have heterotaxic syndromes (abnormal arrangement of the left-right axis) which can be associated with complex congenital cardiac defects [4]. To date there are no specific treatments for PCD, and no evidence based guidelines for clinical management of patients.

‘Better Experimental Approaches to Treat Primary Ciliary Dyskinesia’ (BEAT-PCD; funded by COST Action BM1407) is a network of scientists and clinicians coordinating research from basic science through to clinical care with the intention of developing treatments and diagnostics to improve long-term outcomes for patients (www.BEATPCD.org). Within the first 24 months we have united a multidisciplinary network of 257 participants from 25 countries. Our collaborations have delivered major advances in clinical care, in particular the first evidence-based guidelines for the diagnosis of PCD, and the commissioning of the European Reference Network (ERN) PCD network, part of ERN-LUNG [6]. Step changes have also been made through our research collaborations. For example with BESTCILIA, an international PCD cohort (iPCD) of over > 3000 patients providing infrastructure for epidemiological studies [7] and a PCD registry to support future clinical trials have been established [8], the first multinational clinical trial of a treatment for PCD is analysing results [9], and we have developed, validated and translated quality of life tools (QOL-PCD) for use as outcome measures in trials [10–12].

Workshops at previous BEAT-PCD meetings [13] have identified and prioritised research projects to provide data to support H2020 grant applications. For example BEAT-PCD scientists are conducting a preliminary study to investigate the lung microbiome, and have undertaken a review of pre-clinical models. BEAT-PCD clinicians have identified urgent topics, and formed working group to deliver guidelines for clinical management, e.g. analysing and reporting ciliary ultrastructure, prevention of cross-infection and defining respiratory exacerbations.

This manuscript summarises the 4-day BEAT-PCD Conference meeting and Training school in Valencia Spain in April 2017, including state-of-the-art lectures, oral presentations, workshops and a poster session.

## Work groups activities during the conference and training school

BEAT-PCD activities are coordinated through four integrated work groups: basic science, epidemiology, clinical care and clinical trials (outcome measures). A major role of the basic science work group (WG1) in BEAT-PCD is to share information about advances in science that may translate into clinical research and ultimately clinical practice. PCD is a genetic disease making gene therapy (GT) a potential therapeutic approach. The merits and risks of two such approaches were discussed in plenary talks by Heymut Omran (University of Munster, Germany) and Francisco Dasi (Universitat de Valencia, Valencia, Spain); in addition, a workshop about GT was held during the meeting. Recent advances in imaging technology are transforming biological research, but have not all reached the clinic. Two such approaches (super-resolution (SR) microscopy and electron tomography) in cilia research were discussed by two plenary speakers, Martin Knight (Queen Mary University of London, UK) and Amelia Shoemark (Imperial College London, UK). The role that these technologies may ultimately hold in PCD diagnosis and therapy remains to be seen, but importantly, the clinical community is now more fully aware of the possibilities.

Important challenges in clinical management were discussed during the meeting, led by members of the Epidemiology and Clinical Care Work Groups (WGs 2 & 3). The international PCD registry [8] and the international PCD cohort (iPCD cohort) [7] continue to grow. The iPCD cohort now includes data form over 3200 patients; ongoing analyses look at lung function, diagnostic evaluations, lobectomies and symptoms [14]. New partners can still join and share data from their patients, and already contributing data providers can add data from new patients and add longitudinal data on old patients. The role of a PCD group within the newly developed ERN-LUNG was also presented. The ERNs create a clear governance structure for knowledge sharing and care coordination across EU, to improve access to diagnosis and treatment, as well as the provision of high-quality healthcare for patients with rare diseases [6].

A project within BEAT-PCD WGs 2 and 3 is exploring the delivery of care for PCD across Europe through surveys and interviews with clinicians. This project is relevant because a previous survey, completed almost 10 years ago highlighted disparity across Europe [15]. Another crucial prerequisite for future clinical research is the development of a proforma for standardised prospective collection of clinical data such as symptoms and signs; progress with this project was discussed during the meeting.

Workshops and talks of relevance to clinical care took place (WG3). An expert-led workshop focused on the development of a consensus statement on cross-infections in PCD. Another work shop concentrated on airway clearance. Presentations on the management of the ears and sinuses in patients were made, with a call for clinical trials investigating the use of ventilation tubes (VT).

Very few clinical trials in patients with PCD have been conducted, with only one randomised controlled trial (RCT) being published at the time of the conference [16], and a few clinical trials ongoing. Therefore, WG4 focuses on developing the knowledge and networks for future clinical trials. A challenge to clinical trials is the rarity of the disease. In this regard, Claudia Kuehni (University of Bern, CH) presented how physicians caring for patients with PCD can learn from paediatric oncology, to ensure all patients have the opportunity to participate in research [17]. In order to better understand natural fluctuation of lung function in patients with PCD, Bruna Rubbo (University of Southampton, UK) presented the “Prospective observational multicentre study on variability of lung function in stable PCD patients” (PROVALF-PCD) study. Results from this study will enable a better definition of clinically relevant changes in lung function when new therapies are studied in patients with PCD. Finally, it is of importance for clarification of the effects of different treatments in future pharmaceutical RCTs to establish a functional definition of a clinically significant deterioration or exacerbation in PCD, whether it originates from or is located in the upper or lower respiratory tract, or both. An expert working group met to plan the methodology of a consensus statement for defining pulmonary exacerbations.

## State-of-the-art lectures

Six key note speakers presented on topics relevant to BEAT-PCD. Two presentations concentrated on the potential of gene therapy, and two considered new imaging techniques to advance our knowledge of ciliary structure and function. Further lectures reviewed the similarities and differences between PCD and CF relating to radiological findings and microbiology.

### Gene therapy and PCD

Gene therapy is a term used to describe the technique of repairing or replacing faulty genes in a patient’s cells. However, genetic correction of mutated genes that cause PCD in humans has not yet been successful. In ciliated cell culture models and transgenic mouse models, 3 studies have been published where cilia with PCD mutations were partially restored to normal ciliary beating [18–20]. Lentiviral transfection of wild-type DNAI1 induced normal ciliary movement in cultured human airway cells [18] and in vivo restored approximately 20% of airway ciliary function of a mouse with a mutated Dnaic1 gene [19]. Lai and colleagues used TALENs (transcription activator-like effector endonucleases) to correct mutations in DNAH11, resulting in 29% of the cilia beating normally in cultured human cells [20]. Other gene editing techniques exist, for example CRISPR-Cas9 that can be used in a similar way as TALENs to correct mutations, and collectively, all of these gene replacement or edit therapy techniques show great promise for treating PCD [21]. Heymut Omran spoke about the potential role of gene therapy to treat PCD and discussed the safety concerns of such approaches, highlighting one early gene therapy trial using transgenesis, that resulted in lethal side-effects [22]. He went on to discuss delivering mRNA encoding the affected gene product to the respiratory epithelium, explaining how such an approach could not alter the genome and thus prevent such complications. This approach would however, require regular delivery of the therapy. He also showed research data on delivering mRNA to multi-ciliated cell cultures to rescue PCD phenotypes.

While the ability to correct defective genes causing PCD have been partially demonstrated in-vitro, the big challenges for the future is developing efficient, immunologically safe, targeted delivery systems in humans. With our increasing knowledge of the biology of airway epithelial progenitor cells and their differentiation pathways, one can see that targeting these immature epithelial progenitor cells using techniques such as CRISPR-Cas9 to treat defective PCD genes may prove an excellent target for future gene therapy studies.

### Gene therapy: Lessons from alpha-1 antitrypsin deficiency

Alpha-1 antitrypsin deficiency (AATD) leads, in some patients, to chronic obstructive pulmonary disease (COPD) and liver disease. Francisco Dasi discussed ongoing gene therapy (GT) studies, and trials, that he and colleagues have performed over 30 years to treat this disorder, using transgene insertion into the genome.

He explained that GT is an experimental technique aiming to correct the disease-specific genetic disorders. There are different GT approaches, including the transplantation of the gene to a patient with a deletion, gene correction of the specific mutation, gene augmentation to enhance the expression of gene of interest, or targeted inhibition of gene expression. AATD is caused by mutations in the Serpin Family A Member 1 (SERPINA1) gene, which produces alpha-1 antitrypsin, an enzyme that protects the lungs from the action of neutrophil elastase. There is no cure for the disease. Augmentation therapy is the only approved therapy that has shown some clinical efficacy since it reduces the rate of lung density decline preventing the progression of COPD to more severe stages of the disease. There is no treatment for the liver disease. Alternative strategies currently under investigation include GT. AATD is a good candidate for GT approaches since it is a monogenic disease and the mutations leading to the disease are well-characterized.

He compared between viral vectors (retrovirus, lentivirus, adenovirus, adeno-associated virus, herpes simplex and some others), which take advantage of the natural tropism and the viral capacity for infecting cells; and non-viral vectors, which consist in naked DNA and imply physical procedures (DNA complexes as lipo- and poliplexes, nanoparticles and directed vectors). Finally, he highlighted CRISPR-Cas9 as a revolution in GT.

Based on his and others’ previous experience, Francisco Dasi concluded that GT is a promising approach for a number of diseases, including PCD, because it is a cure for the disease and allows for personalised therapies. However, more work is needed to make sure that it will be safe and effective in patients.

### Super resolution microscopy

Two plenary talks addressed advances in imaging technology. Classic light and fluorescence microscopy is limited by the Rayleigh resolution limit of approximately 200 nm (half the wavelength of light) which corresponds to the width of a cilium [23]. Martin Knight explained how advances in super resolution (SR) microscopy circumvent this limitation, allowing resolution of fixed samples down to ~ 20-40 nm. These methods can improve the visualization of small molecular and cellular structures, providing new understanding of the functioning of cells and their components.

The major group of SR microscopy techniques are so called stochastic switching methods, which use fluorescent molecules (proteins or dyes), which are switched between fluorescence and dark states [24]. Complex statistical analyses of the acquired set of images enables emission of each fluorophore in the image to be very precisely localized from which a SR image is constructed. The resolution limit of these techniques can be similar to transmission electron microscopy (TEM) (~ 20 nm), but there are some technical limitations (e.g. long acquisition time, limited fluorophore selection and imaging depth, fixation of the samples is required) [25, 26]. Examples of these techniques are photoactivated localisation microscopy (PALM); stochastic optical reconstruction microscopy (STORM) or deconvoluted STORM (dSTORM).

Another SR microscopy technique is Structured Illumination Microscopy (SIM). Using SIM, a grid pattern is super-imposed over the specimen, generating an interference pattern. A controlled, stepwise rotation of the grid over the specimen generates a set of images. During image processing, specialized algorithms define the shape of the specimen to be deducted from the patterns. The technique provides a resolution limit of approximately 100 nm, i.e. better than confocal which has a limit of 200 nm but not as good as PALM or STORM [25, 27]. However, in some cases, SIM can allow live cell imaging and its simplicity and ease of use make it an exciting new technique for pushing the limit of resolution. Finally, Martin explained the use of light sheet microscopy that can provide faster, high resolution imaging of living cells or even whole organisms.

Although SR microscopy imaging of non-motile primary cilia or fixed motile cilia has been successfully achieved, the current SR techniques do not allow real-time visualization of ciliary beating in live cells. The resolution limit of SIM might not be enough to visualize complex structures within the ciliary axoneme which will require the use of more resolving SR techniques such as PALM, STORM. Martin Knight concluded that, these are exciting times for cilia imaging with the new development of SR microscopy techniques helping to resolve ciliary structures in live cells or with minimal processing. Although not yet suitable for routine PCD diagnostic use, SR microscopy supports fundamental cilia research and has the potential to help resolve difficult to diagnose cases.

### Novel tools for PCD diagnostics and research: Electron tomography

**Fig. 1:**
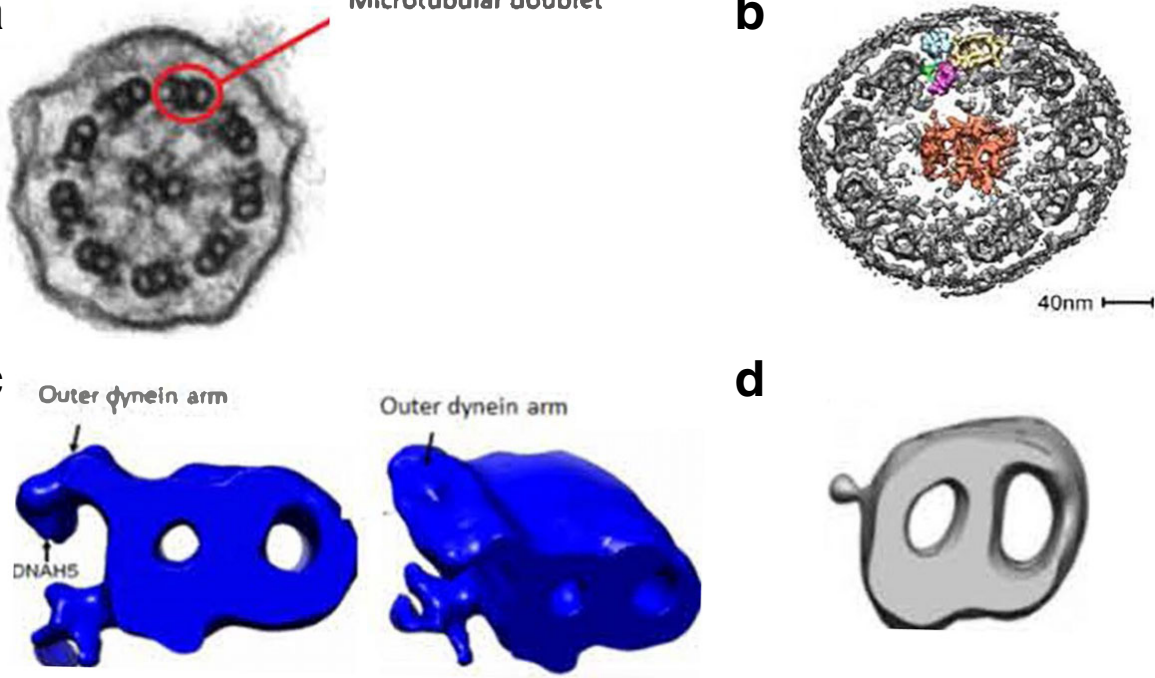
**a** A Transmission electron microscopy image of a cilium in cross section. **b** Electron tomography image of a cilium in cross section with key features coloured (Yellow - microtubular doublet, blue - outer dynein arm, pink - inner dynein arm, green - dynein regulatory complex, orange – central complex). **c** Electron tomogram image of a normal microtubular doublet with improved resolution following computer averaging when compared to the microtubular doublet in image A circled in red. **d** Averaged microtubular doublet tomogram from a subject with PCD due to an inner and outer dynein arm defect

Amelia Shoemark spoke about electron tomography, a technique that allows 3D imaging of cilia at high enough resolution to demonstrate the holes left by proteins that are absent from the cilium [28]. Transmission electron microscopy (TEM) is central to PCD diagnosis and has been used in research for decades (Fig. 1a). Advances to the TEM technique will lead to better understanding of the ciliary structure and ultimately improve diagnosis. The ‘electron tomography’ technique, recently developed (Fig. 1b) is an advanced TEM technique which improves the resolution and spatial visualisation of structures compared to traditional TEM. Rather than a single image, as in TEM, in electron tomography, a series of images of the sample are acquired at different angles. Overlay of all the images generates a tomogram, based on which, a computer 3D model can be generated (Fig. 1c).

Amelia Shoemark started the session by describing the tomography technique and the development of state of the art ciliary electron tomography models by Nicastro et al. [28]. The second half of the session focused on the potential application of electron tomography in PCD diagnostics and gene discovery. 15 to 30% of patients with PCD have normal ciliary ultrastructure when assessed by traditional TEM [2]. This leads to a delay in diagnosis. In many patients who were previously thought to have normal ciliary ultrastructure a structural defect can be demonstrated by electron tomography [29, 30]. For instance, ultrastructural defects were demonstrated in patients with variants in *HYDIN, CCDC164, CCDC65* and *DNAH11* genes where standard TEM is normal. Electron tomography can also be useful in gene discovery, even where a TEM defect is known, through identification of the subtle downstream structural effect of a newly identified PCD gene [31]. The session closed with a discussion around the advantages and disadvantages of the tomography technique.

### PCD is not a variant of CF: Chest imaging

**Fig. 2:**
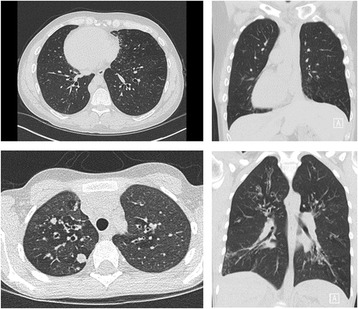
CT images showing dextrocardia and middle lobe abnormalities in adolescent patients with PCD (above), contrasting to upper lobe disease in CF (below)

In the plenary session ‘PCD is not a variant of Cystic Fibrosis’ (CF), Philip Robinson (Royal Children’s Hospital, Melbourne, Australia) summarised the differences between the two conditions with regards to imaging, in particular chest high resolution computed tomography (HRCT) (Fig. 2). He highlighted different patterns of structural change in the two diseases. In PCD neonates with respiratory distress syndrome (RDS), there is a high incidence of lobar collapse, commonly limited to the upper lobes [32]. In infants with CF up to 80% can have an abnormal CT, but RDS and lobar changes are rarely seen; bronchial wall thickening and gas trapping are the commonest findings [33] and there is no predominance for the upper lobes. The pattern of disease seen in infants with PCD is therefore different than in CF and has implications for future lung health. Potential future studies include the long term assessment of PCD patients with neonatal upper lobe disease.

The findings from early childhood contrast to those in older patients where middle and lower lobe disease is more prevalent in PCD, and upper lobe disease more common in CF [34]. Dextrocardia, extensive tree-in-bud patterns, septal thickening and whole lobar collapse are findings seen in patients with PCD. Commonly used HRCT scoring systems [35, 36] have been developed from structural changes characteristic of CF lung disease. As findings in PCD are so different, unique aspects of structural disease could be missed if a CF derived score is directly applied and could limit the description of longitudinal changes and effects of interventions. A collaborative group is developing HRCT scores specifically for PCD, including quantitative outcomes.

### PCD is not a variant of CF: Microbiology

Helle Krogh Johansen (University of Copenhagen, Denmark) delivered a state-of-the-art presentation discussing the similarities and differences in the airway microbiology of PCD and CF. The topic is important because PCD treatment is often directed by CF guidelines. She described the predominance of *Haemophilus influenzae* as a pathogen from the airways of PCD patients, infecting 40–80% of PCD patients annually. *Streptococcus pneumoniae, Moraxella catarrhalis, Staphylococcus aureus, and Burkholderia spp.* are less prevalent but are not rare, and although *Pseudomonas aeruginosa* appears to be less common in children with PCD (approximately 10% in comparison to 30% of CF) it is a common pathogen in adults with PCD*. P aeruginosa* colonises the airway in both conditions, persistence of the same clone has been described for prolonged periods and bacterial adaptation pathways are similar in PCD and CF [37]. PCD microbiology is therefore in many aspects comparable to CF, and in the absence of disease-specific evidence, the speaker argued that it is reasonable to manage infections using CF guidelines, although drug doses may be different.

## Oral presentations

A number of short presentations were made throughout the conference, addressing topics of importance for BEAT-PCD, from basic science and clinical perspectives.

### Basic science oral presentations

Markers of oxidative stress have been detected in exhaled breath condensate of children with PCD [38] and Ana Reula presented her ongoing PhD research investigating oxidative stress in PCD airway epithelium. She analysed reactive oxygen and nitrogen species, glutathione (GSH), and markers of oxidative lipid and protein damage, apoptosis and mitochondria function by flow cytometry in nasal epithelial cells from healthy, PCD and disease control subjects. There was no significant difference in apoptosis, carbonylated proteins, ratio oxidized/reduced lipids, mitochondrial mass and nitric oxide between groups. PCD patients’ airway cells had raised GSH, hydrogen peroxide and total superoxide O_2_^−^ and reduced mitochondrial O_2_^−^. Ana Reula concluded that oxidative stress was implicated in the pathophysiology of PCD and may be dependent on O_2_^−^ and H_2_O_2_ production.

Mahmood Fassad (University College London, UK) spoke about a multigene targeted next generation sequencing approach for novel gene discovery and diagnosis in PCD. A total of 185 PCD patients were studied and a number of novel candidate genes were identified. These now need to be characterised in expression studies using the model multi-ciliated organism *Paramecium*. He went on to emphasise the usefulness of routine genetic testing for PCD diagnosis.

Osman Sezerman has been working closely with Turkish and Japanese populations of patients with aortic aneurisms and performing genome wide association studies to identify single nucleotide polymorphisms within genes. Data was then analysed systematically to look at the biochemical pathways that may be affected by the mutated genes. The data shows that predictable pathways, such as MAP-kinase, were involved. It is thought that this may aid clinical management and diagnosis. Osman Sezerman’s aim is to incorporate multiple clinical phenotypes with genetics for a personalised medicine approach. He suggested that this approach would work well in PCD. However, the complexity caused by consanguinity, and multiple disease causing genes in patients with PCD may complicate the picture.

The intraflagella transport (IFT) *IFT46* gene is thought to be associated with regulation and maintenance of ciliary length during ciliogenesis [39]. Lara Millán presented a study measuring ciliary length in 27 PCD patients’ samples in relation to high-speed video microscopy (HSVM) and TEM data, and *IFT46*, *FOXJ1* and *DNAI2* gene expression. Cilia length was measured in semi-thin TEM sections of airway epithelial samples. 9/27 (33%) patients had normal ciliary length (> 5.9 μm) including 6 patients with normal ultrastructure and function, and 3 with dynein arm defects and immotile cilia. 12/27 (44%) patients had shortened ciliary length (< 5.9 μm) including 3 patients with normal ultrastructure and 9 with dynein arm defects, however all had an abnormal beating pattern. 3/27 (11%) patients had ciliary aplasia and 3 (11%) patients had cilia that could not be measured. Real-time (RT)-PCR showed a 1–2 fold reduction in *FOXJ1* and *DNAI1* gene expression in PCD cases relative to non-PCD controls that were particularly associated with samples that had short cilia or ciliary aplasia. IFT46 expression was reduced in “the short cilia group” but this was not associated with PCD. During questioning, the participants suggested that reduced ciliary length could be due to secondary infection and on-going ciliogenesis therefore further experiments would need to take these potential confounding factors into account.

Male infertility is associated with approximately half of the currently reported PCD genes, yet the effect of these mutations on sperm tail physiology is not understood. Absence of KIF3A in a *Kif3a*^*−/−*^ knockout mouse caused disruption of the manchette organization and abnormal shaping of the sperm head [40] and prevented normal intramanchette of meiosis-specific nuclear structural protein 1, which is required for outer dynein arm assembly within the axonemes of both sperm tail and motile cilia. Loss of *Spef2* gene function in mouse caused a PCD model phenotype [41]. Anu Sironen demonstrated that SPEF2 co-localised with IFT20 and that intramanchette transport of IFT20 was delayed in the absence of functional SPEF2. In conclusion, IFT was required for both sperm tail formation and manchette function and the functional role of different protein isoforms in motile cilia and sperm tail is yet to be fully understood. Anu Sironen proposed *SPEF2* as a potentially new PCD candidate gene and speculated that SPEF2 protein may act as an adaptor for cargo molecules during dynein 1 mediated transport.

### Clinical oral presentations

Antonio Moreno reviewed the literature relating to the definition of pulmonary exacerbations in CF, which may help inform the development of a consensus definition for PCD patients. In clinical practice the physician makes the diagnosis based on changes in signs, symptoms, spirometry, chest radiographs and/ or culture of microbiological samples. However, a more standardised definition is needed where exacerbations are used as an outcome measure for clinical trials. With no consensus definition, recent PCD trials have taken different approaches to defining exacerbations [9, 16], and even in CF there remains no consensus for a single standardised definition [42]. Physician-decision to treat is influenced by many factors and is therefore unreliable as an outcome measure for RCTs; symptom-defined decisions to treat (e.g. Fuchs [43] or modified-Fuchs [42]) is probably better for clinical trials but has not been formally validated; and there is no evidence that scoring systems are reliable or valid [44, 45]. Participants discussed the particular difficulties in defining an exacerbation in PCD, since patients have significant symptoms even when well, and their wet cough does not clear with antibiotics.

Teresa Romero reported a study comparing the broncho-responsiveness to direct and indirect agonists, methacholine, and AMP. Patients with PCD had a higher prevalence of airway hyper-responsiveness (31%) than healthy volunteers (9%) (*p* = 0.06).

Emily Frost reported a study investigating the use of immunofluorescence (IF) labelling of ciliary proteins as a diagnostic test for PCD. The study, which has recently been published, used immunofluorescent labelling of nasal brushings from a discovery cohort of 35 patients diagnosed with PCD by ciliary ultrastructure, and a diagnostic accuracy cohort of 386 patients referred with symptoms suggestive of disease [46]. In the validation cohort IF successfully identified 22 of 25 patients with PCD and staining was normal in all 252 in whom PCD was considered highly unlikely. This indicates that the method is highly specific, but has limited sensitivity and therefore it cannot be used as a stand-alone test.

Heymut Omran updated the conference about ERN-LUNG [6]. The EU is building networks of reference centres to provide quality, expertise, specialism, research and clinical care across Europe. PCD is a core network within the ERN-LUNG. To ensure adequate expertise and experience, PCD reference centres will be expected to care for at least 30 patients, investigate > 70 patients a year using nasal nitric oxide, EM and HSVM and to contribute a minimum dataset to a European PCD registry annually. Nine European centres currently have status as full members of this PCD core network. It is expected that these members will contribute to collectively improving the standard of PCD care across Europe.

Mikkel Alanin discussed chronic rhinosinusitis (CRS) and bacterial sinusitis which affect most patients with PCD, adversely affecting quality of life (QoL). Medical therapies for CRS include nasal irrigation with saline, topical nasal steroids and antibiotics. However, none of these treatments have been formally evaluated in PCD. Endoscopic sinus surgery (ESS) is a well-established CRS treatment in general. Several small, non-randomised studies have evaluated the effect of ESS in PCD and they consistently show benefit [47–49]. Further, ESS can eradicate sinus bacteria and may protect the lower airways from repeated bacterial colonization from the sinuses [47, 50]. Testing the efficacy of CRS treatments in PCD in RCTs is now needed to allow us to develop evidence based guidelines. Otitis media with effusion (OME) affects almost all PCD children causing conductive hearing loss that can impair speech development and QoL. OME may spontaneously improve by age 12 but continues into adulthood in many patients [51]. Ventilation tubes (VT) can restore hearing in patients with OME. However, in PCD use of VT is controversial since VT insertion leads to prolonged otorrhoea in 33–100% of patients [51–53] compared to less than 5% of the general paediatric population. A comparative study on VT indications and efficiency in PCD is highly warranted and an international collaboration between ENT specialists was established during the conference.

## Ongoing and new projects

**Fig. 3:**
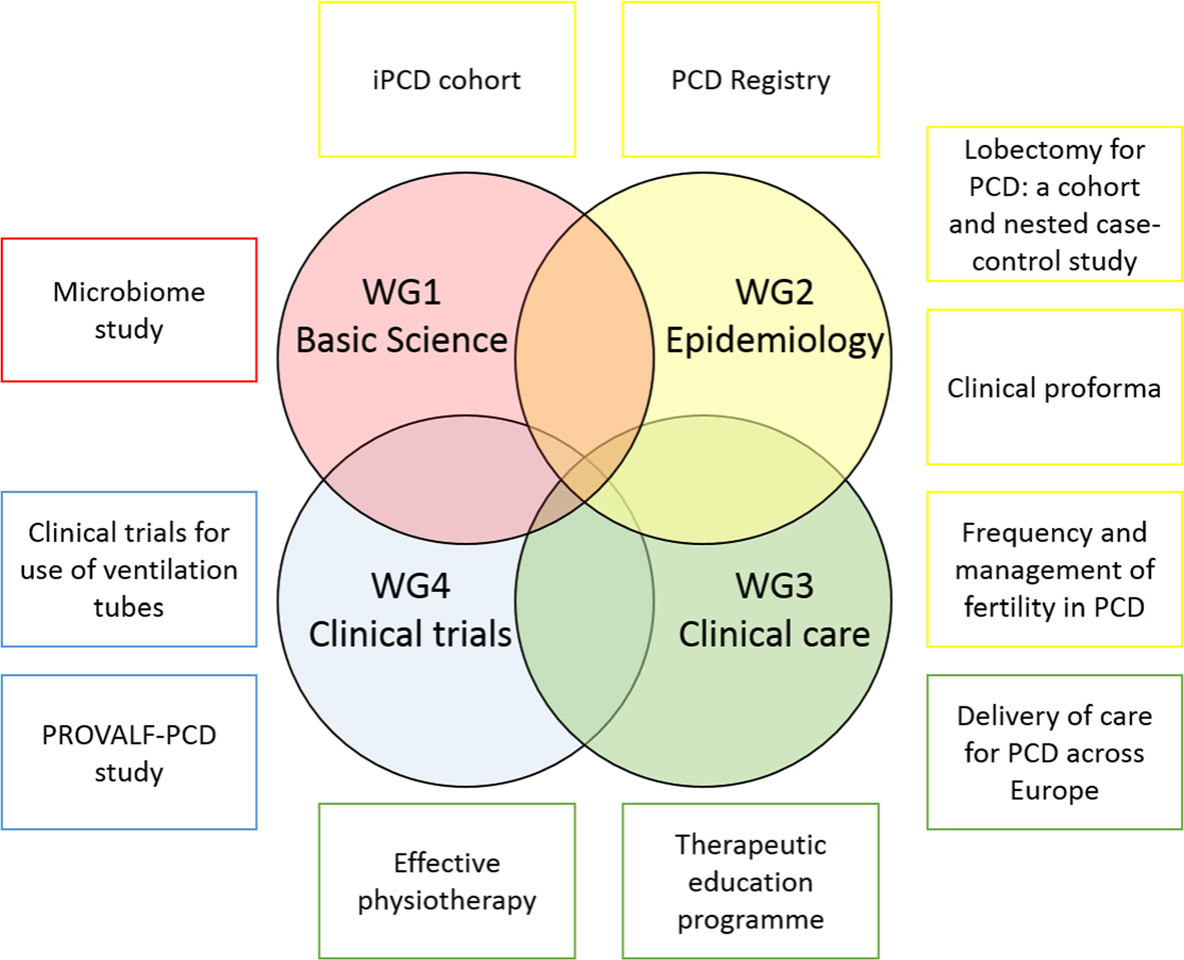
Schematic of ongoing and new projects led by BEAT-PCD members, highlighting their relationship to each of the four work groups (WG)

The Conference highlighted some of the collaborative BEATPCD projects (Fig. 3). Projects are currently in different stages of development, with some more advanced (e.g. publication of iPCD data) while others still in development (e.g. protocol development). More details on the projects and their current status are included in Additional file 1.

In addition to the updates about ongoing projects, Claudia Kuehni gave a talk on “Clinical studies in PCD: can we learn from Paediatric Oncology” discussing how we can use experiences from paediatric oncology to improve recruitment to PCD projects [17]. The following actions were crucial in the success of improving treatment in paediatric oncology:

- Centralized care: patients are only treated in a few specialised clinics, which collaborate together in a network, and there are strict standards for all centres;
- Central registration: all patients are registered in clinical or population-based national registries and data is pooled internationally for research;
- Standardised care: care for patients is standardised on national and international level;
- Clinical studies: almost all patients are invited to participate in one or more clinical trials.

Claudia Kuehni compared the situation in PCD with paediatric oncology 30–40 years ago, with many malignancies occurring at very low prevalence and varying practices of follow-up and treatment of childhood cancer. Internationally recognized study protocols then changed the picture. All children diagnosed with childhood cancer were ultimately treated according to the respective protocols. This led to a dramatic increase in survival over the last 40 years, better understanding of the disease and of different treatment options as well as near 100% participation of these patients in clinical trials. In contrast to childhood cancer, for which mortality is a well-defined outcome marker, different possible outcome parameters for studies in patients with PCD need validating – lung function being one of the most important ones.

## Training school workshops

A variety of workshops were designed to address the training needs of a multidisciplinary network at differing stages of career.

### Airway clearance workshop

Gemma Marsh and Victoria Keenan facilitated a workshop on airway clearance. The aims of this workshop were:

- To introduce the concepts of upper and lower airway clearance in PCD
- To share and discuss the UK National PCD Service Physiotherapy guidelines (unpublished)
- To bring together physiotherapists from different countries, with the aim of sharing practice and developing a network to guide/design future research projects.

The workshop was divided into two sections, an oral presentation and a practical workshop. The facilitators introduced concepts of both upper and lower airway clearance techniques currently utilised in the UK. The oral presentation was designed to be interactive and create discussion using multimedia (videos, pictures); it related research evidence and clinical experience to underpin practice. This was followed by an interactive practical session. Attendees had opportunity to practice a number of techniques and equipment, and to discuss the pros, cons and practicalities of each.

Developing a network of PCD physiotherapists is essential in order to share practice and develop research opportunities. Currently this network is lacking and given the nature of rare diseases, this can mean therapists are working in non-specialist areas or in isolation. In the spirit of BEAT-PCD this strengthening of infrastructure and building links for therapists for clinical support, experience and knowledge unites the aim to improve patient experience and outcomes.

### Transition workshop

A workshop on transition between paediatric and adult services was hosted by Amanda Harris and Amanda Friend. An effective transition between paediatric and adult services improves the long term outcomes of young people with chronic health needs, improving morbidity and mortality [54–56]. In practice there is often confusion between ‘transition’ and ‘transfer’ with the education and empowerment of young people being overlooked. This interactive workshop aimed to:

- Explore the differences between transition and transfer
- Discuss how practices differ across Europe, focussing on common challenges and good practice
- Introduce the “Ready, Steady, Go” (RSG) programme as a tool to aid transition
- Discuss how to improve outcomes for children and young people with PCD going into adult services

Morbidity and mortality has been shown to increase following transfer from paediatric to adult services for many different diseases [57–61]. A planned and purposeful transition programme, individualised around the young person can improve the experience and outcomes of the young people [54–56]. Transition is a process of education and empowerment slowly moving the emphasis from the parent to their child ensuring that on transfer, the young adult is equipped with the skills and knowledge they need to manage their condition. It seeks to prepare the young person for adult services rather than preparing the adult service to receive the young person [62]. Transfer is a single event when care is passed from paediatric to the adult services.

The RSG programme is a simple but comprehensive tool to help guide transition. It is a holistic programme encompassing all aspects of daily life allowing the young person to guide their clinic appointments to focus on the issues that matter to them. RSG ensures that the medical, psychosocial, and vocational needs of the young person are addressed following a structured adaptable transition programme [62]. Starting the process early, around the time of transfer to senior/high school, allows plenty of time to deliver the programme in bite size chunks.

This interactive workshop allowed much discussion around the differences in health care provision and cultures across the different countries and its impact on the transition process for young people. Many common challenges and successes were expressed. Transition arrangements differed across Europe but overall the RSG programme was seen as a good tool that would serve to guide transition in most situations.

### Gene therapy workshop

Francisco Dasi and Daniel Pellicer hosted a workshop on gene therapy (GT). The term “Gene Therapy” was coined many years ago by researchers speculating on the possibility of correcting specific genetic defects by introducing external genetic material to correct the defective gene and potentially getting a cure for inherited monogenic diseases. From the beginning, it became clear that the main challenge to GT, before it could be used into the clinical practice, was the construction of safe and efficient vectors that allowed the delivery of the therapeutic genetic material. Three decades later, some of these problems remain unsolved, however, in recent years there have been advances that indicate promising results for the use of GT in daily clinical practice.

Significant advances have been made in the genetic aspects of PCD, which has led to the identification of several genes that could be candidates for the different GT techniques. Since there is currently no treatment for PCD, GT could be a therapeutic alternative (similar to that already existing in other rare respiratory diseases) that would potentially lead to a cure.

The facilitators’ used their experience from AATD gene therapy, to provide awareness of the basic concepts of GT which might be translated into PCD research. Daniel Pellicer gave the initial talk, where the following items were introduced and discussed:

- Basic concepts of replacement GT and gene editing;
- The progress made since the initial steps of GT and the problems encountered and still unresolved;
- The different strategies used for gene transfer (in vivo vs. ex vivo and viral vs. non-viral).

Following this theoretical discussion, there was a practical part in which participants were asked to split into two groups with the purpose that each group developed its own GT project in PCD. Participants developed a 10 min presentation in which they explained their research projects. Both groups chose to use systems of gene editing and non-viral gene therapy. Finally, in a final 15 min discussion, both projects were discussed and special emphasis was placed on the challenges (choosing the appropriate candidate gene; delivery problems; how to overcome undesired immune responses and how to avoid off-target effects), the ethical aspects and the commercial viability of GT in PCD.

The final conclusion was that, despite several hurdles that need to be overcome, GT is potentially a curative treatment that could be used for the treatment of PCD. In addition, the technology could generate patents and would therefore be of interest to the pharmaceutical companies. Since some of the members of the BEAT-PCD group have previous experience in GT and others in the development of experimental models of the disease, we proposed the possibility of using these models to evaluate the efficiency of the GT techniques in the treatment of the disease.

### High-speed video microscopy analysis workshop

BEAT-PCD delegates attended a workshop on HSVM analysis hosted by Claire Jackson, Robert Hirst and Ana Reula. The workshop focussed on the role and methods of HSVM within the diagnostic pathway in the context of the recently published European Respiratory Society Diagnostic Guidelines [2, 63]. The work group discussed the need for expertise, a standardised approach, specialised equipment [64]. It was recommended that centres should develop their own normative data since ex vivo ciliary function varies depending on environmental conditions, yet standardised methods are not in place; for example some centres measure ciliary function at 37 °C, whilst other conduct analyses at room temperature, factors such as pH and culture medium might also influence results.

The facilitators emphasised the importance of taking samples when patients have been free of an upper respiratory infection for at least 4 weeks to minimise the likelihood of secondary defects. Ciliary beat frequency (CBF) is a quantitative measure of ciliary function which can be calculated by manual [65] or computational analysis [66, 67], however CBF without ciliary beat pattern (CBP) analysis misses cases of PCD when CBF is within ‘normal range’. The group discussed the subjective nature of CBP analysis and the potential for objective parameters to be measured [67].

Workshop participants reviewed examples of normal ciliary movement with mucociliary clearance, and then examples of cilia that were immotile, rotating, stiff, staggered and unsynchronised. It was emphasised that more subtle abnormalities of ciliary movement could easily be missed by HSVM [68]. In conclusion, HSVM was shown to be a valuable diagnostic method when conducted and interpreted correctly.

## Consensus statement workshops

The Inaugural BEAT-PCD Conference in 2015 had identified topics for consensus statements [13]. Three of these topics were taken forward during Workshops of experts during this Conference. A consensus statement to define pulmonary exacerbations was considered important for future clinical trials; consensus guidelines for the prevention of cross-infections between patients was also considered a priority; and the European Respiratory Society Guidelines for diagnosing PCD had called for standardisation of testing and reporting of TEM [2].

Twenty pulmonologists and a nurse specialist formed the Working Group to define pulmonary exacerbations; Siobhan Carr and Jane Lucas invited a pulmonologist from each COST country represented at the conference. A short presentation reviewed definitions used in CF and non-CF bronchiectasis. The Workshop members decided that a definition of pulmonary exacerbation was needed for research purposes rather than for clinical care, to enable standardised use across clinical trials. It was agreed that a modified Delphi e-survey would be used to reach consensus; we also decided to invite more adult pulmonologists, some physiotherapists and patient representatives to join the working group.

Kim Nielsen and Helle Krogh Johansen convened a multidisciplinary, multinational working group to develop a consensus statement for PCD and cross-infections. There were considerable differences within the group concerning present practices in out-patients, in-patients and for patients attending meetings. It was agreed that there are similarities between CF and PCD microbiology, and in the absence of evidence to the contrary cross-infection between patients is a significant risk. The group decided to develop a consensus guideline for publication, working electronically until meeting at next year’s conference.

Amelia Shoemark and Estelle Escudier invited 12 PCD electron microscopy experts representing 9 countries to meet in person and via video link to plan a consensus statement for reporting ciliary ultrastructure by TEM. European diagnostic guidelines recommend ‘a hallmark defect’ at TEM as a confirmatory diagnostic test for PCD [2]. However, there is currently no international classification of ultrastructure for cilia biopsies and there is considerable heterogeneity among pathology reports describing PCD. Differences in reporting can result in difference in interpretation of findings between centres. In recent years advances in genetic testing and molecular biology have redefined many aspects of the disease further complicating the use of traditional reporting terminology. For example the use of the term radial spoke defect has been used to describe both central complex defects and microtubular disarrangement defects [69–71]. In renal allograft pathology, internationally agreed pathology classification known as ‘the Banff criteria’ [72] has been shown to improve disease outcome. The aim of this workshop was to form a working group to provide an internationally agreed ultrastructural classification for PCD diagnosis [72].

Three objectives for the classification statement were formulated and agreed at the meeting:

- To define hallmark defects diagnostic for PCD
- To describe features which should be included in a TEM report to assist multidisciplinary diagnosis of PCD or exclusion of the condition
- To define adequacy of a diagnostic sample

During the TEM workshop, experts created a list of features which could be included in a pathology report. The inclusion of these items will be considered by Delphi consensus survey by 16 members, representing 16 institutions worldwide, to form the guideline. The first draft of the consensus guideline will be completed by September 2017. The guideline will then be tested and updated by the group prior to meeting at the next BEAT-PCD conference.

## Short term scientific missions

STSMs provide an excellent training resource with the opportunity to visit a research institution in another country, allowing access to resources and expertise not available in the participants’ institution. The aim is that early stage researchers (ESRs) will use the knowledge and skills acquired during the STSM to advance PCD research at their institution and develop collaborative projects supporting the aims of BEAT-PCD. STSMs can also be used to invite an expert to a developing PCD centre to provide on-site training and expertise. Up to 15 bursaries are awarded each year. More information on STSM applications can be found on the BEAT-PCD website (http://www.BEAT-PCD.org/).

During this Conference and Training School, STSM participants presented overviews of their experience during their visit.

Ana Reula, University of Valencia, spent 3 months at the University of Southampton UK, hosted by Jane Lucas’ group. She gained experience in the preparation and analysis of diagnostic test samples for HSVM, TEM and IF. In particular, she learnt to perform culture of respiratory epithelium at air-liquid interface (ALI), and sample preparation and analysis of ciliated ALI-cultures. She attended multidisciplinary diagnostic meetings, PCD research meetings and had the opportunity to visit the Royal Brompton Hospital, London, to observe the diagnostic pathway at another UK centre.

Myrofora Goutaki, University of Bern CH, visited the ENT clinic of the Kremlin-Bicetre Hospital, Paris France, supervised by Jean-François Papon. She observed the diagnostic, follow-up and management protocols of PCD patients, observed interviews, clinical examinations and nasal functional measurements and got an overview of the local PCD databases. She identified important questions of clinical relevance for the ENT management and follow-up of PCD patients and discussed ongoing projects and development of future collaborative project with members of the French multidisciplinary PCD team.

Raquel Jacinto from the Nova Medical School (Lisbon, Portugal) visited the lab of Mauro Pistello at the University of Pisa, Italy. She learned cell transfection skills, which she has applied to building new zebrafish PCD mutants in her laboratory for a collaborative project testing CRSPR-Cas9 gene editing for mutating PCD genes in parallel systems: the zebrafish and ex vivo human respiratory nasal epithelia.

Mieke Boon and Martine Jaspers, Leuven Belgium, visited Heymut Omran’s lab in Munster Germany, to learn the technical challenges of ALI culture and IF antibody staining. Research collaboration was enhanced with the exchange of genetic analysis techniques and implementation of the patient registry for research and clinical applications.

## Poster session

A key objective of BEAT-PCD is to promote involvement of ESRs in research and all the activities of BEAT-PCD. The poster session of the Conference and Training School demonstrated the success of PCD research by ESRs (Table 1: list of posters; Part 2: poster abstracts). The posters came from a broad range of disciplines: epidemiologic studies, clinical research and basic science projects, reflecting the multidisciplinary nature of the BEAT-PCD network. Topics ranged from a study of the QoL of patients with PCD, to the assessment of novel methods for the diagnosis of the disease. The session included also a wide range of important basic science projects from microbiology to genetic studies. The poster session attracted senior clinicians, scientists and young researchers allowing an excellent opportunity for cross pollination of ideas and potential future collaborations.

**Table 1:** Details of 16 posters from 9 countries, presented at the 2nd BEAT-PCD Conference & 3rd PCD Training School (only listed if authors agreed to have the title published)

Poster title	Authors (Country of first author)
Development of a bioinformatics analysis method for diagnosis of Primary Ciliary Dyskinesia	Alik E (Turkey)
Quality of life in patients with primary ciliary dyskinesia: a systematic review	Behan L, Rubbo B, Lucas JS, Dunn Galvin A (Ireland & UK)
Estimation of cell number in a three-dimensional cell cluster – comparison of different 3D nuclei calculating methods.	Kraft M, Bukowy-Bieryllo Z, Fedoruk-Wyszomirska A, Dabrowski M, Wyszko E, Pikulska M, Witt M, Zietkiewicz E (Poland)
Diagnosing Primary Ciliary Dyskinesia (PCD) using electron microscopy and exome sequencing	Crowley S, Shoemark A, Kulseth MA, Reinholt F, Heimdal K (Norway)
In vitro differentiation of respiratory epithelial cells in the sequential culture model	Dabrowski M, Bukowy-Bieryllo Z, Wyszomirska-Fedoruk A, Pikulska M, Witt M, Zietkiewicz E (Poland)
Preliminary Results of Whole Exome Sequencing in Turkish Primary Ciliary Dyskinesia Patients- Hacettepe University Experience: “Three candidate genes, five novel and two known mutations”	Emiralioğlu N, Taşkıran E, Koşukçu C, Tuğcu GD, Eşref S, Hızal MG, Yalçın E, Ersöz DD, Kiper N, Alikaşifoğlu M, Özçelik U (Turkey)
Antibiotic resistance of Haemophilus influenza in Primary Ciliary Dyskinesia Patients	Çıkı K, Demirci S, Sancak B, Emiralioğlu N, Tuğcu GD, Eşref S, Hızal MG, Yalçın EG, Ersöz DD, Kiper N, Şener B, Özçelik U (Turkey)
Changes in height and BMI in children and adolescents with Primary Ciliary Dyskinesia: an iPCD Cohort study	Goutaki M, Halbeisen F, Maurer E, Casaulta C, Crowley S, Haarman E, Lucas JS, Morgan L, Nielsen KG, Santamaria F, Schwerk N, Thouvenin G, Yiallouros P, Latzin P, Kuehni CE (Switzerland)
Lung growth in children and young adults with Primary Ciliary Dyskinesia: an iPCD Cohort study	Halbeisen FS, Goutaki M, Maurer E, Casaulta C, Crowley S, Haarman E, Lucas JS, Morgan L, Nielsen KG, Santamaria F, Schwerk N, Thouvenin G, Yiallouros P, Zivkovic Z, Latzin P, Kuehni CE (Switzerland)
The effect of L-Arginine on Cilia Beat Frequency in PCD patients, non-PCD referrals and healthy controls	Kouis P, Hadjisavvas A, Middleton N,Papatheodorou S, Kyriacou K, Yiallouros PK (Cyprus)
The role of Rabconnectin3a in cilia length regulation	Tavares B, Pestana S, Lopes S (Portugal)
Late diagnosis of the PCD is still a great risk	Martinu V, Dvorakova P, Uhlik J, Pohunek P (Czech Republic)
Quality of life questionnaire for Spanish patients with Primary Ciliary Dyskinesia	Reula A, Behan L, Pastor S, Castillo S, Bañuls S, Navarro-García MM, Lucas JS, Dasi F, Escribano A, Armengot M (Spain)
Ten years’ experience of Primary Ciliary Dyskinesia Diagnostic Testing	Rubbo B, Behan L, Lima de Queiroz AP, Goggin P, Jackson C, Packham S, Walker W, Lucas JS (UK)
A high prevalence CCDC103 p.His154Pro mutation causing primary ciliary dyskinesia is associated with normal diagnostic investigations	Shoemark A, Moya E, Hirst RA, Patel MP, Robson EA, Hayward J, Scully J, Fassad MR, Lamb W, Schmidts M, Dixon M, Patel-King RS, Rogers AV, Rutman A, Jackson CL, Goggin P, Ollosson S, Carr S, Walker W, Adler B, Loebinger MR, Wilson R, Bush A, Williams H, Boustred C, Jenkins L, Sheridan E, Chung EMK, Watson CM, Cullup T, Lucas JS, Kenia P, O’Callaghan C, King SM, Hogg C, Mitchison HM (UK)
Use of electron tomography to confirm the diagnosis of primary ciliary dyskinesia	Shoemark A, Burgoyne T, Kwan R, Cahill T, Scully J, Dixon M, Patel M, Bush A, Mitchison H*, Hogg C* (UK)

## Difficult case discussions: Diagnostic testing

This interactive session on diagnostic dilemmas was new to the BEAT-PCD programme and proved very popular and informative. Chaired by Claire Hogg and Kim Nielsen, participants were encouraged to bring their diagnostic dilemmas in the form of clinical history, including screening tests and other investigative pathway details. Ciliary function (HSVM clips) and ultrastructure (TEM) and any other advanced diagnostic tests such as IF, genetics or 3D electron tomography were then viewed to allow for the assembled field of experts to share their opinions and own similar experiences.

Cases presented included:

- Deborah Snijders (Italy) presented a case where TEM had been done multiple times and showed a central complex defect - All agreed this was PCD. She highlighted that in their diagnostic guidelines TEM for central complex defects is not considered ‘hallmark’ in these cases making it difficult to officially confirm.
- Andreia Pinto (Portugal) showed a TEM only case. She did not have the clinical details for this case but presented multiple TEM views for the panel. Estelle Escudier and Amelia Shoemark agreed that there was an absence of outer dynein arms. This conclusion was confirmed by other experts in the room highlighting the importance of holding such a multidisciplinary meeting.
- Claire Hogg (UK), showed 2 cases:
  - A series of unusual TEM findings showing interconnections between the ciliary axonemal cross sections. The axonemal ultrastructure was otherwise normal. These cases had a high prevalence of asthma/atopy phenotypes, but also had low or borderline low nasal NO. No-one else had seen these unusual appearances, but a fruitful discussion about possible aetiology and possible staining methods to determine their structure was held.
  - A case of very subtle ODA defects at the tip of the cilia with some sections apparently having normal ultrastructure. This partial defect was subsequently found to be caused by a DNAH5 mutation. Experience was shared about these more subtle defects and the role of extended testing in all difficult cases to help solve them was debated.
- Carmen Casaulta (Switzerland) presented a clinical case with typical phenotype but normal TEM. Her centre did not have access to HSVM, and so the discussion centred on the valuable role of additional testing, HSVM in particular, where typical cases have normal TEM. DNAH11 mutations was discussed as a likely mutation and it was suggested that in addition to investigating the cilia beat pattern IF, genetics and electron tomography could be considered.

The session ended with a discussion about developing networks for discussion of difficult diagnostic dilemmas. This is something that exists in an informal setting where larger and more developed centres review cases from time to time, with the added provision of training. However, further discussion about developing a formal network is warranted.

## Difficult case discussions: Clinical management

The management of PCD patients remains challenging, in the main, because there is a limited evidence base to guide clinicians on best practice. To date there have only been two RCTs in PCD.

One tested hypertonic saline and did not demonstrate a significant difference in the primary outcome measure between treatment and placebo groups (St Georges Respiratory Questionnaire) [16], but did report differences in one of the sub-domains of the secondary outcome measure (Quality of Life Bronchiectasis questionnaire QOL-B).. The other assesses the effect of azithromycin, and results are not yet available [9]. Hence management is based on expert opinion, consensus guidelines, and extrapolated from the larger evidence base available for patients with CF. Though it has many similarities to PCD, CF clearly has a different pathophysiology and applying the same treatment strategies from CF to PCD may not only be ineffective but potentially detrimental [73]. Discussion of difficult clinical cases of PCD is therefore key to further developing expert consensus on management. Four such challenging cases were presented during this interactive session.

Cases presented included:

- Suzanne Crowley (Norway), described a patient clinically diagnosed with PCD and managed as having PCD prior to the availability of PCD diagnostic testing. 16 years later these investigations were undertaken and found to be normal, highlighting the importance of PCD diagnostic reference centres to be available to confirm diagnoses.
- Woolf Walker (UK), described a teenager with PCD and a severe respiratory phenotype (FEV_1_ 15% predicted, oxygen dependent, on overnight BiPAP support with end stage bronchiectasis) but, despite this a good perceived quality of life. The case highlighted the limited international experience on lung transplant in PCD patients to help inform decision making in such severe patients.
- Nisreen Rumman (Palestine), described a 17-year-old diagnosed late with PCD despite having typical clinical features since birth and a deceased brother with lung disease. The patient had a rapidly progressive course despite aggressive management. The case highlighted the lack of knowledge about PCD among general practitioners that had led to the delay in diagnosis and the difficulty of access to diagnosis and some medications in resource limited countries.
- Zorica Zivkovic (Serbia), described a 9-year-old with sinopulmonary disease and ear infections who was being treated as an atopic case; however PCD investigations demonstrated immotile cilia. The case highlighted the importance of keeping high index of suspicion for PCD since the symptoms are non-specific.

These cases and the discussions that followed, highlighted both the difficulties in diagnosing and managing these complex patients but also the disparities and challenges in care provision across different countries.

## Evaluation feedback from participants

**Fig. 4:**
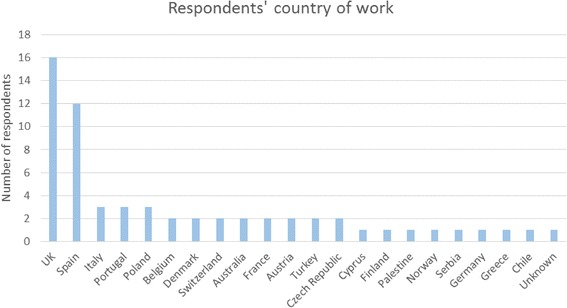
2nd BEAT-PCD Conference feedback survey respondents’ country of work

**Fig. 5:**
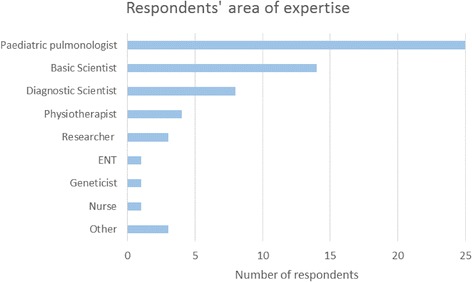
2nd BEAT-PCD Conference feedback survey respondents’ area of expertise

**Fig. 6:**
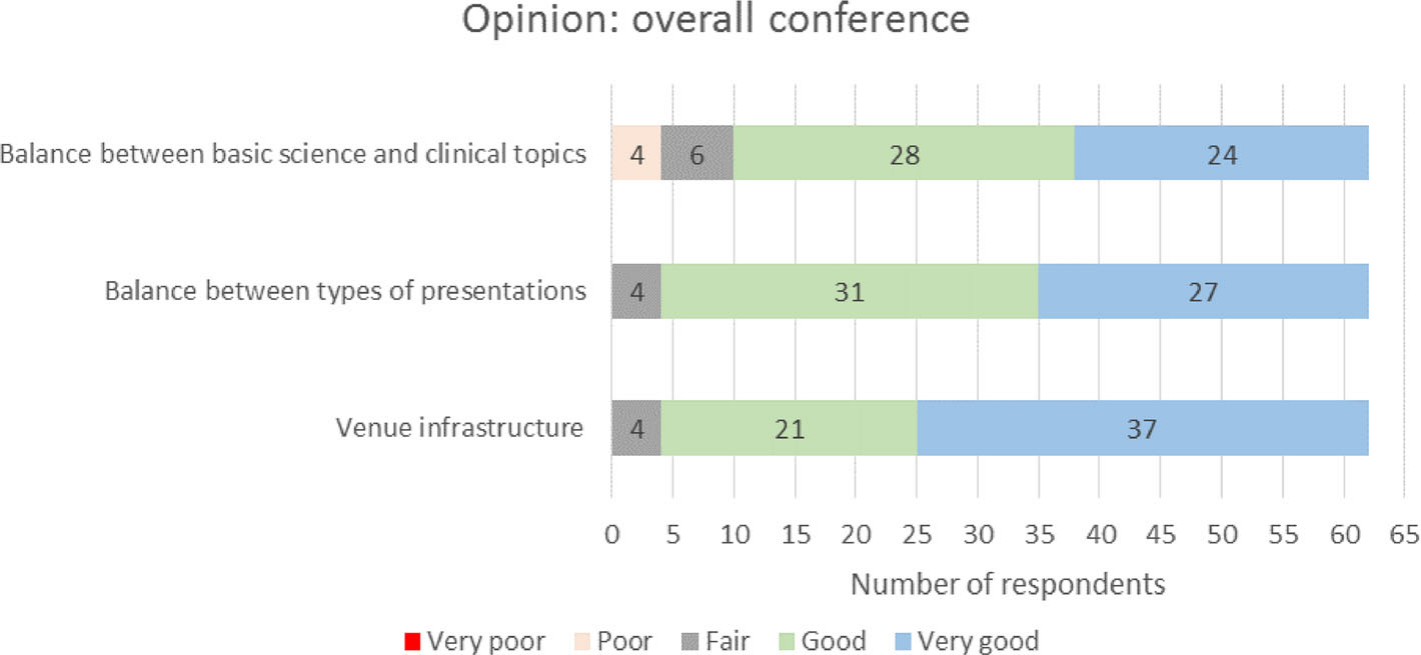
Feedback survey respondents’ opinion on the structure of the 2nd BEAT-PCD Conference

An online survey was circulated to participants during the Conference and Training School for feedback and to improve subsequent BEAT-PCD Conferences. Sixty-two participants from 21 countries (Fig. 4) responded, of which 47% ESRs. Participants from diverse multidisciplinary backgrounds (Fig. 5) evaluated the selection of topics and the time allocated to each of the conference sessions (Fig. 6).

Participants rated the overall conference 8.9 out of 10, with 90% stating that their needs were fully addressed independent of career stage. Plenary sessions, workshops, and opportunities for networking and discussing new and ongoing projects were highlighted as the most useful aspects of the conference. The majority of participants (93%) approved this year’s event format: a joint Conference and Training School, with oral presentations in the morning followed by interactive sessions (workshops, difficult cases) in the afternoon.

## Summary

The BEAT-PCD Conference and training school brought together clinical PCD specialists (paediatricians and adult pulmonologists, ENT, physiotherapists, specialist nurses) and scientists from varied backgrounds (genetics, imaging, cell biology, microbiology, bioinformatics). The multidisciplinary conference provided an interactive platform for research groups from 21 countries to exchange ideas through a program of lectures, poster presentations, breakout sessions and workshops. The next BEAT-PCD conference and training school will be held in Lisbon, Portugal in February 2018.

## Additional file

Additional file 1: Aims, methods, outcomes and current status of ongoing and new BEAT-PCD projects, presented at the 2nd BEAT-PCD Conference & 3rd Training School. (DOCX 30 kb)

## Abbreviations

AATD: Alpha-1 Antitrypsin Deficiency
CBF: Ciliary beat frequency
CBP: Ciliary beat pattern
CF: Cystic Fibrosis
COPD: Chronic obstructive pulmonary disease
CRS: Chronic rhinosinusitis
CT: Computed tomography
ERN: European Reference Networks
ERN-LUNG: European Reference Network for rare respiratory conditions
ESRs: Early Stage Researchers
ESS: Endoscopic sinus surgery
GSH: Glutathione
GT: Gene therapy
H2O2: Hydrogen peroxide
HSVM: High-speed video microscopy analysis
IF: Immunofluorescence
IFT: Intraflagella transport
iPCD: International PCD cohort
MNS1: Meiosis-specific nuclear structural protein 1
NO: Nitric oxide
OME: Otitis media with effusion
PCD: Primary ciliary dyskinesia
QoL: Quality of life
RCT: Randomized controlled trials
RNS: Reactive nitrogen species
ROS: Reactive oxygen species
RSG: Ready, Steady, Go
SIM: Structured Illumination Microscopy
SR: Super-resolution
STSMs: Short-Term Scientific Missions
TALENs: Transcription activator-like effector endonucleases
TEM: Transmission electron microscopy
VT: Ventilation tubes
