# Supplementary material for: Proceedings of the 2nd BEAT-PCD conference and 3rd PCD training school: part 1
Source: BMC Proc. 2018 Mar 5;12(Suppl 2):1. doi: 10.1186/s12919-018-0098-9 (PMC5841193; doi:10.1186/s12919-018-0098-9)
Supplement: Supplementary file 1 — Aims, methods, outcomes and current status of ongoing and new BEAT-PCD projects, presented at the 2nd BEAT-PCD Conference & 3rd Training School. (DOCX 30 kb) [file 12919_2018_98_MOESM1_ESM.docx]

**Additional file 1** Aims, methods, outcomes and current status of ongoing and new BEAT-PCD projects, presented at the 2^nd^ BEAT-PCD Conference & 3^rd^ Training School

| **Project title** | **Lead country** | **Aims** | **Methods** | **Current status** |
| --- | --- | --- | --- | --- |
| Microbiome study | UK | - Investigate the interaction between use of antibiotics and microbiota | Prospective observational study | Protocol development |
| International PCD (iPCD) cohort | Switzerland | - Assemble all available datasets on PCD worldwide to answer pertinent questions on clinical phenotype, disease severity, prognosis and effect of  treatments | Retrospective cohort study | Several ongoing (data analysis and manuscript preparation) and planned projects;  Dataset enrichment: new centres join, minimise missing data and update diagnostic data |
| PCD Registry | Germany | - Develop a disease-specific registry to prospectively collect data | Prospective longitudinal study | Data collection |
| Clinical proforma | Switzerland | - Develop a standardised proforma for patient follow-up | Adapted Delphi process to develop final proforma and pilot clinical proforma in outpatient clinics | Adapted Delphi approach to finalise proforma |
| Lobectomy for PCD: a cohort and nested case-control study | Cyprus | - Describe prevalence of lobectomies in PCD patients and compare characteristics between lobectomised and non-lobectomised PCD patients | Longitudinal retrospective study (nested in the iPCD cohort) | Manuscript preparation |
| Frequency and management of fertility problems in patients with PCD | France | - Describe the prevalence and the characteristics of fertility problems in adult male and female patients with PCD  - Describe the percentage of ectopic pregnancies in PCD  - Investigate the current management of fertility problems in PCD | Multi-centre longitudinal prospective study, international survey and patient-based questionnaires | Proforma development |
| Delivery of care for PCD across Europe | UK | - Investigate differences in service delivery methods  - Describe ‘essential resources’ for development of new centres | Mixed methods research using questionnaires and semi-structured interviews with PCD specialists, and an international survey | Interviews and data coding |
| Therapeutic education programme | France | - Assist patients to acquire self-care and adaptive competencies  - Determine the level of understanding by parents and children on disease management  - Develop educational tools | Qualitative interviews by multidisciplinary team followed by educational intervention | Implementation of the therapeutic educational programme tools across France |
| Effective physiotherapy | UK | - Identify physiotherapists working with PCD patients across Europe to develop network for future communication  - Compare physiotherapy practices | Discussions between physiotherapists across Europe | Network development |
| PROVALF-PCD study | UK, Switzerland & France | - Determine the natural variability of lung function measurements in stable PCD patients | Prospective longitudinal study using routinely collected data and spirometry every 3 months | Study-specific database development |
| Clinical trial on use of ventilation tubes in PCD | France | - Determine if ventilation tubes or hearing aids improve hearing outcomes and quality of life for PCD children  - Describe disadvantages and acceptability of treatment options | Clinical trial | Protocol development |
